# Systemic inflammatory response index as a novel biomarker for age-related macular degeneration: a cross-sectional study from NHANES (2005–2008)

**DOI:** 10.3389/fnut.2025.1540933

**Published:** 2025-03-06

**Authors:** Ruoshuang Jia, Yiqing Yin, Huimin Shan

**Affiliations:** ^1^Department of Anesthesiology, Tianjin Medical University Cancer Institute and Hospital, Tianjin, China; ^2^Department of Ophthalmology, Beijing Chaoyang Hospital, Capital Medical University, Beijing, China

**Keywords:** association, systemic inflammatory response index, risk, age-related macular degeneration, NHANES

## Abstract

**Background:**

Chronic low-grade systemic inflammation plays a significant role in age-related macular degeneration (AMD) pathogenesis. The systemic inflammatory response index (SIRI), a novel inflammatory marker, may predict various diseases. However, data on the relationship between SIRI and AMD are limited. This study examines the relationship between SIRI and AMD and assesses its potential as a predictive biomarker.

**Methods:**

A cross-sectional analysis of the National Health and Nutrition Examination Survey (NHANES) data from 2005 to 2008 was conducted on participants aged ≥40 years with SIRI and AMD status data. Multivariable logistic regression models adjusted for confounders were used to assess the association. Sensitivity and subgroup analyses, along with restricted cubic spline (RCS) curve analysis, were performed.

**Results:**

Among 5,365 participants, 425 (7.9%) had AMD. The median SIRI was higher in AMD patients (1.23 vs. 1.04, *p* < 0.001). Higher SIRI was independently associated with increased odds (adjusted OR: 1.18, 95% CI:1.07–1.29, *p* = 0.001). RCS analyses revealed a dose–response relationship (*p* = 0.002). Subgroup analyses showed a positive association in male participants, individuals with hypertension, individuals with obesity, and non-smokers. Higher SIRI levels were independently associated with increased AMD risk (adjusted OR: 1.27, 95% CI: 1.03–1.56, *p* = 0.023).

**Conclusion:**

Elevated SIRI is independently associated with increased AMD risk in the U.S. population. SIRI may serve as a biomarker for identifying high-risk individuals, enabling early intervention. The cross-sectional design limits causal inference, and unmeasured confounders may affect the results. SIRI could potentially serve as a non-invasive biomarker for AMD risk, pending further validation through longitudinal studies.

## 1 Introduction

Age-related macular degeneration (AMD) is a progressive, inflammatory retinal disorder that primarily affects the macula (1), leading to severe vision impairment. Symptoms include difficulty performing tasks in low light or low contrast, visual distortion, and reduced visual acuity (2). AMD has become a significant public health concern due to the growing aging population and increasing life expectancy (3, 4). Globally, approximately 196 million people are affected by AMD (5). It is a leading cause of severe vision loss in individuals aged >55 years in high-income countries, and it imposes a significant psychosocial burden (6, 7). Late-stage AMD manifests as either geographic atrophy (dry AMD) or neovascularization (wet AMD), with 6–9% of cases leading to legal blindness worldwide (6). Established risk factors include age, genetic factors, and environmental factors (5).

The pathogenesis of AMD is complex and multifactorial, involving genetic susceptibility, aging-related disruption of retinal homeostasis, impaired lipid metabolism, immune activation, chronic inflammation, oxidative stress, and extracellular matrix (ECM) dysfunction (1). Among these factors, chronic inflammation plays a significant role. It promotes drusen formation, which can lead to severe vision loss (8). In AMD, inflammation is triggered by the adaptive immune response to accumulated molecular damage in the aging eye, leading to local immune activation (9). Chronic inflammation and tissue damage are associated with abnormal activation of the complement system (10, 11). Chronic inflammation also recruits microglia and macrophages to the subretinal and choroidal regions—key indicators of immune activation in AMD (8). Additionally, senescent cells resulting from chronic inflammation secrete pro-inflammatory cytokines and chemokines, further activating microglia, macrophages, and the complement system, contributing to the inflammatory response (8, 12).

Chronic inflammation induces oxidative stress (1), which damages retinal neurons and the retinal pigment epithelium (RPE), worsening inflammation and tissue damage (13, 14). It is also associated with lipid deposition and oxidation in drusen, generating pro-angiogenic signals that contribute to choroidal neovascularization, which is a late-stage manifestation of AMD (1, 15). Furthermore, chronic inflammation disrupts retinal homeostasis, impairing ECM maintenance and clearance of cellular debris, both of which contribute to AMD progression (16, 17). In summary, chronic inflammation is a central feature of AMD pathogenesis, contributing to tissue damage, immune system activation, and disease progression.

Early detection of AMD can leverage inflammatory biomarkers such as elevated interleukin-6, C-reactive protein, and white blood cell levels (18–22). Systemic inflammatory markers are advantageous for disease prediction due to their low cost and accessibility. They have recently gained popularity for predicting several diseases, including AMD (23–27). The systemic inflammatory response index (SIRI), identified by Qi et al. in 2016, is a novel marker that quantifies inflammation using peripheral blood parameters (28). It has been proposed as a predictor for various conditions, including cancer, cardiovascular diseases, and pneumonia (29–41). However, its relationship with AMD prevalence remains unclear. We hypothesize that higher SIRI levels are positively associated with AMD risk, given the role of systemic inflammation in AMD pathophysiology. Unlike neutrophil-to-lymphocyte ratio (NLR) and platelet-to-lymphocyte ratio (PLR), which are linked to other inflammatory conditions, SIRI may better capture a broad systemic inflammatory response by including lymphocyte, neutrophil, and monocyte counts. This population-based study aimed to investigate the association between SIRI and the risk of AMD.

## 2 Materials and methods

### 2.1 Participants

This cross-sectional study primarily utilized data from the National Health and Nutrition Examination Survey (NHANES), a widely used dataset for analyzing the nutritional and health status of individuals in the United States. Data for this study were collected from 2005 to 2008. The analysis included 5,365 participants aged ≥40 years with valid data on SIRI and AMD status.

### 2.2 Outcome variable

The primary outcome variable was AMD status, defined based on the standardized grading of retinal photographs. AMD was detected through the presence of (i) soft drusen and pigmented abnormalities, (ii) geographic atrophy or pigment epithelial detachment, (iii) subretinal hemorrhage or visible subretinal new vessels, and (iv) subretinal fibrous or laser treatment scars (42, 43).

### 2.3 Exposure variable

SIRI was calculated using the formula:

$\mathrm{SIRI}=\left(\mathrm{monocyte}\;\mathrm{count}\times \mathrm{neutrophil}\;\mathrm{count}\right)/\mathrm{lymphocyte}\;\mathrm{count}$ as defined in previous studies (28).

### 2.4 Covariates

Covariates included demographic data (age, sex, ethnicity, and marital status), medical history (diabetes and hypertension), body mass index (BMI), obesity, and smoking status. Detailed information on variable collection methods is available in the NHANES Survey Methods and Analysis Guide. Age is a well-established risk factor for AMD, with its prevalence increasing significantly over time (4, 44). Women may have a higher risk due to hormonal factors and longer life expectancy (45). Racial and ethnic disparities exist, with Non-Hispanic White individuals at the highest risk (46). Marital status may influence health behaviors and access to healthcare, indirectly affecting AMD risk (47). Diabetes and hypertension contribute to AMD risk through mechanisms involving chronic inflammation, oxidative stress, and vascular health (48, 49). Obesity, linked to systemic inflammation and metabolic dysfunction, may exacerbate AMD development (50). Smoking is a well-documented risk factor due to its role in oxidative stress and inflammation (51, 52). These covariates were selected to account for potential confounders and ensure a comprehensive analysis of AMD risk determinants.

### 2.5 Data analysis

Baseline variables were characterized based on AMD presence. Continuous variables were expressed as mean ± standard deviation, while categorical variables were presented as numbers (%). Differences between groups were analyzed using analysis of variance for continuous variables and chi-square tests for categorical variables.

Multivariable logistic regression models were used to assess the association between SIRI and AMD risk, adjusting for potential confounders. SIRI was also categorized into tertiles based on the distribution of SIRI values in our study population to evaluate linear trends between SIRI and AMD risk. In the following extended models: (i) model 1 was unadjusted; (ii) model 2 was adjusted for sex, age, marital status, and race/ethnicity; and (iii) model 3 was further adjusted for obesity, hypertension, diabetes, and smoking status. A restricted cubic spline (RCS) curve was used to explore the relationship between SIRI and AMD risk.

Subgroup analyses were conducted with stratified factors comprising (i) sex (male/female), (ii) race (Mexican American, Non-Hispanic White, Non-Hispanic Black, or Other Races), (iii) hypertension (yes/no), (iv) diabetes (yes/no), (v) obesity (yes/no), and (vi) smoking status (non-, former, or current smokers).

Sensitivity analyses were conducted to assess the stability of the results. Participants with SIRI scores below the 5th percentile or above the 95th percentile were excluded, and multivariate logistic regression analyses were re-conducted.

Statistical significance was set at *p* < 0.05, and all analyses were conducted using R software (version 4.1.1).

## 3 Results and discussion

### 3.1 Participant characteristics

A total of 5,365 participants (male (n) = 2,687 (50.1%), female (*n*) = 2,678 (49.9%)) with a median age of 59 years were included in the study. Among them, 425 (7.9%) participants had AMD. Participants with AMD were more likely to be older, Non-Hispanic White, of other marital status, and to have lower BMI, hypertension, and a history of former smoking compared to those without AMD (all *p* < 0.05). Additionally, the median SIRI was significantly higher among participants with AMD compared to those without AMD (1.23 vs. 1.04, *p* < 0.001). Table 1 presents participant characteristics categorized by AMD status.

**Table 1:** Baseline characteristics according to the age-related macular degeneration.

Characteristics	Total (*n* = 5,365)	Non age-related macular degeneration (*n* = 4,940)	Age-related macular degeneration (*n* = 425)	*p* value
**Age (years), median [IQR]**	59.00 [49.00, 69.00]	58.00 [48.00, 67.00]	73.00 [64.00, 80.00]	<0.001
**Gender**				0.502
Female	2,678 (49.9)	2,473 (50.1)	205 (48.2)	
Male	2,687 (50.1)	2,467 (49.9)	220 (51.8)	
**Ethnicity, *n* (%)**				<0.001
Mexican American	839 (15.6)	787 (15.9)	52 (12.2)	
Non-Hispanic Black	1,043 (19.4)	1,008 (20.4)	35 (8.2)	
Non-Hispanic White	2,928 (54.6)	2,625 (53.1)	303 (71.3)	
Other Races	555 (10.3)	520 (10.5)	35 (8.2)	
**Marital status, *n* (%)**				<0.001
Married	367 (6.8)	352 (7.1)	15 (3.5)	
Never married	3,226 (60.1)	3,007 (60.9)	219 (51.5)	
Other	1772 (33.0)	1,581 (32.0)	191 (44.9)	
**Hypertension, *n* (%)**				<0.001
No	2,424 (45.2)	2,291 (46.4)	133 (31.3)	
Yes	2,941 (54.8)	2,649 (53.6)	292 (68.7)	
**Diabetes, *n* (%)**				0.452
No	4,162 (77.6)	3,839 (77.7)	323 (76.0)	
Yes	1,203 (22.4)	1,101 (22.3)	102 (24.0)	
**BMI, mean (SD)**	29.26 (6.47)	29.33 (6.53)	28.44 (5.60)	0.007
**Obesity, *n* (%)**				0.006
No	3,321 (61.9)	3,031 (61.4)	290 (68.2)	
Yes	2044 (38.1)	1909 (38.6)	135 (31.8)	
**Smoking status, *n* (%)**				<0.001
Never	2,546 (47.5)	2,357 (47.7)	189 (44.5)	
Former	1732 (32.3)	1,560 (31.6)	172 (40.5)	
Now	1,087 (20.3)	1,023 (20.7)	64 (15.1)	
SIRI (10*9/L), (median [IQR])	1.05 [0.72, 1.50]	1.04 [0.71, 1.48]	1.23 [0.83, 1.81]	<0.001
**Tertile of SIRI, *n* (%)**				<0.001
Tertile 1	1790 (33.4)	1,685 (34.1)	105 (24.7)	
Tertile 2	1787 (33.3)	1,662 (33.6)	125 (29.4)	
Tertile 3	1788 (33.3)	1,593 (32.2)	195 (45.9)	

Values are presented as *n* (%), median [IQR] or mean (SD). IQR, interquartile range; SD, standard deviation; SIRI, systemic inflammatory response index.

### 3.2 Association between SIRI and AMD

The association between SIRI and AMD risk was assessed through multivariable logistic regression analyses (Table 2). When SIRI was analyzed as a continuous variable, a positive correlation with AMD was observed (*p* < 0.001). This positive correlation persisted after adjusting for potential confounders (*p* = 0.001).

**Table 2:** Associations between the SIRI and age-related macular degeneration risk in the total cohort.

SIRI	Cases with AMD/N	Model 1^a^		Model 2^b^		Model 3^c^	
		OR (95%CI)	*p* value	OR (95%CI)	*p* value	OR (95%CI)	*p* value
Tertile categories							
Tertile 1 (0.060–0.826)	105/1790	1		1		1	
Tertile 2 (0.827–1.318)	125/1787	1.21 (0.92–1.58)	0.169	1 (0.76–1.32)	0.977	0.98 (0.75–1.3)	0.912
Tertile 3 (1.319–20.500)	195/1788	1.96 (1.53–2.51)	<0.001	1.44 (1.11–1.87)	0.006	1.36 (1.05–1.77)	0.021
P for trend		<0.001		0.001		0.006	
Continuous values (0.060–20.500)	425/5365	1.34 (1.22–1.47)	<0.001	1.21 (1.1–1.33)	<0.001	1.18 (1.07–1.29)	0.001

a Model 1 was crude model and adjusted for nothing.

b Model 2 was adjusted for age, gender, marital status, race.

c Model 3 included the covariates of Model 2 with additional adjustment for obesity, hypertension, diabetes, smoking status.

SIRI, systemic inflammatory response index; OR, odds ratio; CI, confidence interval.

**Figure 1:**
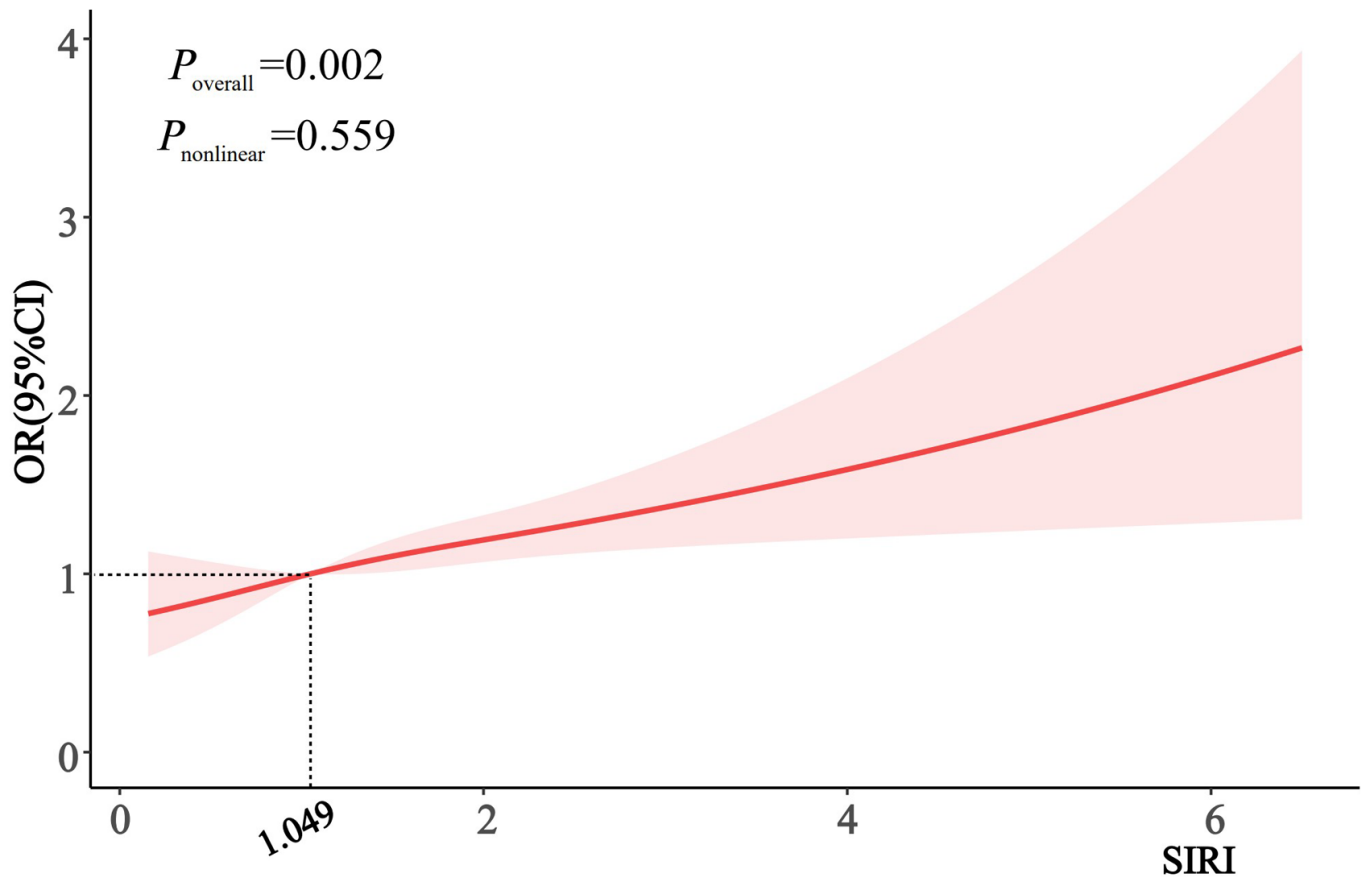
Dose-response correlation between SIRI and AMD risk.

When SIRI was analyzed as a categorical variable, in model 3, participants in the highest SIRI tertile had an increased risk of AMD prevalence compared with those in the lowest SIRI quartile (*p* = 0.021). This association remained statistically significant across all three models. However, the second SIRI tertile did not exhibit a significant association with AMD risk in model 3. The trend test indicated a statistically significant association between SIRI and AMD risk (*p* for trend =0.006). Furthermore, RCS analyses demonstrated a dose–response relationship between SIRI and AMD risk (*p* = 0.002; Figure 1).

AMD is an age-related retinal disorder affecting the macula, with inflammation playing a pivotal role in its pathogenesis. Various cytokines and immune cells, including macrophages, dendritic cells, neutrophils, T-lymphocytes, and B-lymphocytes, contribute to both innate and adaptive immune responses involved in AMD progression (53). Elevated SIRI may reflect increased neutrophil-driven oxidative stress and monocyte-mediated chronic inflammation, both key contributors to AMD progression. Oxidative damage can initiate inflammation and lead to AMD-like lesions, as demonstrated in models where oxidative fragments elicit immune responses mimicking AMD pathology (54). Elevated NLR, a marker of systemic inflammation, has been linked with AMD, particularly its neovascular subtype (55). Activation of toll-like receptors (TLRs) in the RPE induces pro-inflammatory responses, contributing to chronic inflammation and AMD progression (56, 57). Additionally, genetic polymorphisms in complement system components, such as complement factor H (CFH), are strongly associated with AMD, with overactive complement pathways driving inflammation and disease progression (58, 59).

These findings suggest that inflammatory markers and cytokines could serve as potential diagnostic biomarkers for AMD (29). Already, anti-inflammatory therapies targeting specific pathways, such as the complement system and TLRs, are being explored as treatment options (53, 56). Thus, inflammation in AMD involves complex interactions between oxidative damage, immune responses, and genetic predispositions, offering potential opportunities for diagnostic and therapeutic strategies aimed at mitigating inflammation and managing AMD.

SIRI has been proposed as a predictor for various diseases, including cancers, cardiovascular diseases, and pneumonia (29–41). However, research examining its association with AMD risk remains limited. The existing literature presents conflicting perspectives on the relationship between leukocytes and AMD. While some studies suggest that elevated white blood cell counts are associated with an increased risk of AMD (22, 60), others report no significant correlation (52, 61). Recently, the use of various blood components from routine complete blood count (CBC) measurements to detect inflammatory markers in patients with AMD has garnered attention. For example, patients with AMD have been shown to have higher neutrophil and lower lymphocyte levels compared to controls (55, 62, 63). Furthermore, Karahan et al. reported that patients with wet AMD have significantly higher NLR and monocyte-to-lymphocyte ratio than those with dry AMD and healthy controls (64).

These studies highlight the potential usefulness of CBC parameters as markers of AMD-associated inflammation. Neutrophils, the most abundant type of leukocytes, play a significant role in both acute and chronic inflammation by mediating phagocytosis and releasing anti-inflammatory mediators (65, 66). Lymphocytes are involved in the onset and resolution of inflammation, responding to different activation or inhibition signals. Lymphocyte infiltration is associated with the initiation and progression of inflammatory responses, contributing to tissue damage and functional impairment in inflammatory diseases (66, 67). Similarly, monocytes contribute to inflammation (66, 68). Elevated PLR, often linked to thrombocytosis-induced lymphocytopenia, has been associated with poor prognoses in inflammatory disorders (69, 70). Among these leukocyte subtypes, neutrophils and lymphocytes appear to have a more significant role in AMD pathogenesis. Consequently, including other leukocyte types in SIRI calculations may detract from its usefulness as a focused inflammatory marker.

The results of the present study indicated that SIRI had a significant positive correlation with AMD risk. A recent retrospective study (71) reported no significant difference in SIRI scores between the AMD and control groups, suggesting that SIRI may be insufficient to assess AMD-related inflammation. However, this study had a major limitation, which was its small sample size (case group (*n*) = 90, control group (*n*) = 270), which limited its statistical power to identify certain trends or correlations. In contrast, the present study utilized a much larger sample of 5,365 participants and found a marked positive relationship between SIRI and AMD prevalence.

### 3.3 Subgroup analysis

Subgroup analyses stratified by sex, ethnicity, hypertension, diabetes, obesity, and smoking status were conducted to investigate the relationship between SIRI and AMD in different populations (Table 3). The degree of association varied across subgroups.

**Table 3:** Association between SIRI and AMD in different subgroups.

Subgroup	OR (95%CI)	*p*-value
Gender		
Male	1.48 (1.01–2.15)	0.043
Female	1.21 (0.83–1.76)	0.32
Ethnicity		
Mexican American	0.94 (0.48–1.86)	0.863
Non-Hispanic Black	1.47 (0.67–3.24)	0.336
Non-Hispanic White	1.61 (1.15–2.26)	0.006
Other Races	1.29 (0.52–3.2)	0.586
Hypertension		
No	1.2 (0.76–1.89)	0.438
Yes	1.47 (1.06–2.04)	0.02
Diabetes		
No	1.32 (0.98–1.78)	0.069
Yes	1.65 (0.93–2.92)	0.084
Obesity		
No	1.27 (0.92–1.75)	0.146
Yes	1.64 (1.02–2.62)	0.04
Smoking status		
Never	1.71 (1.14–2.57)	0.009
Former	1.18 (0.77–1.8)	0.45
Now	1.06 (0.57–1.97)	0.85

OR, odds ratio; CI, confidence interval.

SIRI was positively associated with AMD risk among male participants (*p* = 0.043). Similarly, significant associations were observed among participants with hypertension (*p* = 0.020), obesity (*p* = 0.040), and non-smokers (*p* = 0.009). These findings suggest that the association between SIRI and AMD risk was statistically significant in most subgroups. However, some subgroups did not exhibit significant association.

Previous studies have consistently shown that smoking significantly increases the risk of AMD and its subtypes. In comparison, current smokers had a significantly increased—approximately two-to three-fold—risk of AMD compared to non-smokers (51, 72–75), with geographic atrophy (GA) and neovascular AMD showing varying degrees of statistical significance in different studies (72, 74). Additionally, the AMD risk increases with cumulative smoking exposure, with individuals exceeding 40 pack years having a significantly higher risk (74). Genetic interactions, such as those involving the CFH and *LOC387715* genes, further increase AMD risk in smokers, suggesting that individuals with certain genetic predispositions have higher risks when they smoke (76–79). Quitting smoking reduces this AMD, with former smokers showing a gradual decline in AMD prevalence over time (73–75). Smoking contributes to AMD pathogenesis through oxidative stress and exacerbation of ocular inflammation, both of which damage retinal tissues (78, 80).

The present study’s findings indicated that individuals with AMD demonstrated a higher probability of lower BMI compared to those without AMD. However, the positive association between SIRI and AMD was more pronounced in participants with obesity. A meta-analysis (81) including 1,613 individuals from MEDLINE, EMBASE, and ISI Web databases reported a 32% increased risk of late AMD in obese participants (relative risk [RR]: 1.32, 95% CI: 1.11–1.53, *p* < 0.01), while no significant association was found for early AMD (RR: 0.91, 95% CI: 0.74–1.08; *p* = 0.67). These differences may be due to the varying impacts of obesity across different AMD stages. Additionally, the association between SIRI and AMD appeared stronger in males than in females, potentially due to higher antioxidant gene activity and enzyme levels in females, which could mitigate oxidative stress and reduce AMD risk (82, 83).

This study also revealed a statistically significant association between SIRI and AMD in participants with hypertension. Similar to previous studies, participants with AMD were more likely to develop hypertension than those without AMD (84, 85). Hypertension-related oxidative stress, pro-inflammatory factors, and other pathological mechanisms may underlie this relationship (86, 87). These findings emphasize the usefulness of SIRI for assessing inflammation in patients with hypertension, enabling early detection and prevention of AMD.

### 3.4 Strengths and limitations

This study has several strengths. First, to the best of our knowledge, this is the first study to investigate the association between SIRI and AMD in a general population. Second, the use of the NHANES database, which has a large and representative sample that allows for the exclusion of possible interfering factors, enhances the reliability of the study’s findings. Third, by focusing on SIRI as a composite metric instead of its components, this study offers a comprehensive understanding of the complex interactions between inflammatory processes and AMD.

Despite its strengths, this study has several limitations. First, the cross-sectional design precludes the establishment of causal relationships between SIRI and AMD. Second, self-reported medical conditions introduce the potential for recall bias. Further prospective studies are needed to address these issues by clarifying the exact relationship between SIRI and AMD. Third, although multiple confounders were excluded, the influence of unmeasured confounders cannot be entirely ruled out. Finally, due to limitations in the NHANES dataset, this study focused on eligible adults, leaving gaps in understanding SIRI’s association with AMD in high-risk older populations. However, explanations on the association of SIRI with AMD in high-risk older populations are not provided because the usefulness of SIRI for detecting AMD is crucial in this demographic.

## 4 Conclusion

This study involved cross-sectional analyses of data from 5,365 individuals from the NHANES database to investigate the association between SIRI and AMD risk. The findings demonstrated a positive association between SIRI and AMD prevalence. Participants in the highest SIRI tertile demonstrated significantly higher odds of AMD compared to those in the lowest tertile, even after adjusting for potential confounders. Additionally, higher SIRI scores significantly increased the risk of AMD among males, participants with obesity, non-smokers, and those with hypertension. Overall, the findings demonstrated that elevated SIRI is independently associated with an increased risk of AMD in the US population.

These findings suggest that SIRI could be used as a biomarker for AMD risk assessment, which can greatly benefit patient care. Since it is derived from peripheral blood, its measurement is straightforward, cost-effective, and non-invasive. This makes it highly suitable for widespread use in routine screening, especially in primary healthcare settings. Such routine screening can lead to early diagnosis and intervention, which are key to improving the prognosis of AMD patients. For instance, closer eye monitoring can be arranged to detect any early-stage symptoms of AMD. Lifestyle changes, such as dietary adjustments and increased physical activity, can be recommended to mitigate the risk factors associated with AMD development. Additionally, preventive therapies, like antioxidant supplementation or anti-inflammatory medications, can be initiated. All these measures have the potential to either slow down the progression of AMD or, in the best-case scenario, prevent its onset altogether. This not only improves the quality of life for patients but also reduces the economic burden associated with long-term AMD management.

However, it is crucial to acknowledge the limitations of this study. The cross-sectional design of this study precludes the establishment of a causal relationship between SIRI and AMD. Although an association between them exists, it is possible that other unmeasured factors contribute simultaneously to the elevation of SIRI and the development of AMD.

To address the current knowledge gaps, further prospective studies are urgently needed. These studies should track large cohorts over an extended period, precisely measure SIRI levels at regular intervals, and closely monitor the development of AMD. By doing so, they can confirm the current results and, more importantly, explore the causal relationship between SIRI and AMD. Additionally, future research should focus on clarifying how the components of SIRI, such as the ratios of different immune cells, relate to the pathogenesis of AMD. This in-depth understanding will be instrumental in developing more targeted and effective prevention and treatment strategies for AMD, ultimately benefiting a large number of patients worldwide.

## Data Availability

The original contributions presented in the study are included in the article, further inquiries can be directed to the corresponding author.
